# Molecular diagnosis of anti-laminin 332 (epiligrin) mucous membrane pemphigoid

**DOI:** 10.1186/s13023-018-0855-x

**Published:** 2018-07-06

**Authors:** Roxana Chiorean, Sorina Danescu, Oana Virtic, Mayson B. Mustafa, Adrian Baican, Annette Lischka, Takashi Hashimoto, Yoshinobu Kariya, Manuel Koch, Cassian Sitaru

**Affiliations:** 1 Department of Dermatology, University Medical Center Freiburg, Hauptstrasse 7, 79104 Freiburg, Germany; 2 Department of Dermatology, University of Medicine and Pharmacy “Iuliu Hatieganu”, Cluj-Napoca, Romania; 3 Department of Dermatology, Osaka City University Graduate School of Medicine, Osaka, Japan; 4 Department of Biochemistry, Fukushima Medical University, Fukushima, Japan; 5 Institute for Dental Research and Oral Musculoskeletal Biology and Center for Biochemistry, Medical Faculty, University of Cologne, Cologne, Germany

**Keywords:** Autoantibody, Autoimmunity, Autoantigen, ELISA, Extracellular matrix, Immunoassay, Immunobloting

## Abstract

**Background:**

Mucous membrane pemphigoid is a group of chronic subepithelial autoimmune blistering diseases that mainly affect mucous membranes. Laminin 332-specific autoantibodies are present in approximately 1/3 of the patients, being associated with an increased risk of malignancy. Because of the severe complications, an early recognition of the disease allowing a timely therapy is essential. The gold standard methods for detection of laminin 332-specific autoantibodies, including the immunoprecipitation and immunoblotting are non-quantitative, laborious and restricted to a few specialized laboratories worldwide. In addition, the use of radioimmunoassays, although highly sensitive and specific, are laborious, expensive and tightly regulated. Therefore, there is a stringent need for a quantitative immunoassay for the routine detection of laminin 332-specific autoantibodies more broadly available to diagnostic laboratories. The aim of this study was to compare different antigenic substrates, including native, recombinant laminin 332 and laminin 332-rich keratinocyte extracellular matrix, for development of an ELISA to detect autoantibodies in mucous membrane pemphigoid.

**Results:**

Using a relatively large number of sera from MMP patients with well-characterized autoantibody reactivity we show the suitability of ELISA systems using laminin 332 preparations as adjunct diagnostic tools in MMP. While glycosylation of laminin 332 does not appear to influence its recognition by MMP autoantibodies, ELISA systems using both purified, native and recombinant laminin 332 demonstrated a high sensitivity and good correlation with the detection of autoantibodies by immunoblotting. ELISA systems using different laminin 332 preparations represent a feasible and more accessible alternative for a broad range of laboratories.

**Conclusions:**

Our findings qualify the use of immunoassays with the laminin 332-rich preparations as an ancillary diagnostic tool in mucous membrane pemphigoid.

**Electronic supplementary material:**

The online version of this article (10.1186/s13023-018-0855-x) contains supplementary material, which is available to authorized users.

## Background

**Fig. 1:**
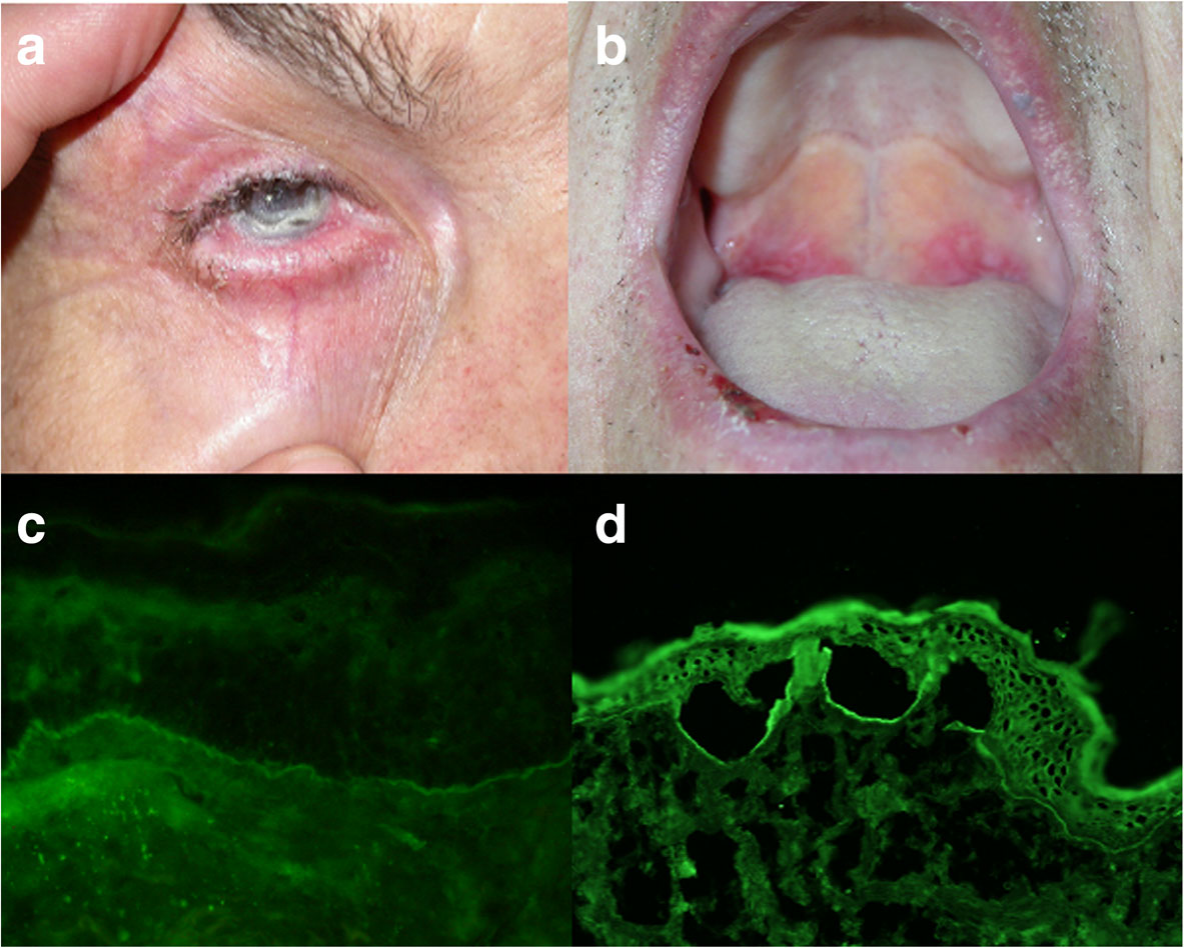
Characteristic clinical and immunofluorescence findings in patients with anti-laminin 332 mucous membrane pemphigoid. **a** Buccal erosionsin a 80 year-old male patient. **b** Ocular involvement in an 80 year-old male patient. **c** Direct immunofluorescence microscopy shows IgG deposits at the dermo-epidermal junction of a patient with anti-laminin 332 mucous membrane pemphigoid. **d** Serum IgG autoantibodies binding to the dermal side of 1 M NaCl-split skin by indirect immunofluorescence microscopy

Mucous membrane pemphigoid (MMP) represents a group of chronic, inflammatory, subepithelial autoimmune blistering diseases that predominantly affect mucous membranes and potentially also the skin, leading to progressive scarring and significant functional impairment [1, 2]. The most commonly affected sites are the oral and ocular mucosae (Fig. 1a, b) [3]. MMP has typically a smouldering course, occassionaly under the clinical form of low-grade mucosal diseases, such as chronic conjunctivitis, desquamative gingivitis, periodontal disease, with rather slow progression to often irreversible, severe complications, including esophageal, anal stenosis, urethral strictures and blindness [1]. Skin involvement occurs in one third of MMP patients and manifests itself as a chronic vesiculobullous eruption, including the variant designated as Brunsting-Perry pemphigoid [1].The diagnostic work-up, prompted by a high index of suspicion, relies on different laboratory investigations, including the histopathological analysis, the detection of tissue-bound immunoreactants by direct immunofluorescence microscopy as well as of serum autoantibodies by indirect immunofluorescence microscopy and molecular immunoassays [1, 4]. Diagnosis is confirmed by the detection of IgG and/or IgA and C3 deposits at the epithelial basement membrane by direct immunfluorescence (IF) microscopy of the perilesional mucosa or skin and the detection of circulating autoantibodies against the epithelial basement membrane by indirect IF microscopy or molecular immunoassays [1, 5].

The rather low clinical MMP activity is paralleled by a relatively low autoantibody reactivity in peripheral blood of the patients. Not unexpectedly, in up to 50% of MMP patients no autoantibodies are detected by indirect IF microscopy on salt-split skin [1, 4]. In roughly two thirds of the MMP patients, their IgG and/or IgA autoantibodies target collagen XVII/BP180, while approximately one third show autoantibodies to laminin 332 [1, 6–9]. Further autoantigens described in MMP of lower prevalence and/or diagnostic significance, include BP230 [10], α_6_β_4_ integrin [11] and collagen VII [12, 13].

Pathogenicity of laminin 332-specific autoantibodies has been demonstrated by the passive transfer of rabbit IgG or Fab fragments against laminin 332 into neonatal mice [14, 15, 18]. Patients having the MMP variant associated with laminin 332 (epiligrin)-specific autoantibodies show an increased risk to develop malignancies, usually solid cancers [16]. Because of the severe complications, an early recognition of the disease is mandatory, allows the timely initiation of the immunosuppressive therapy, and justifies an extensive tumour screening [17, 18].

**Fig. 2:**
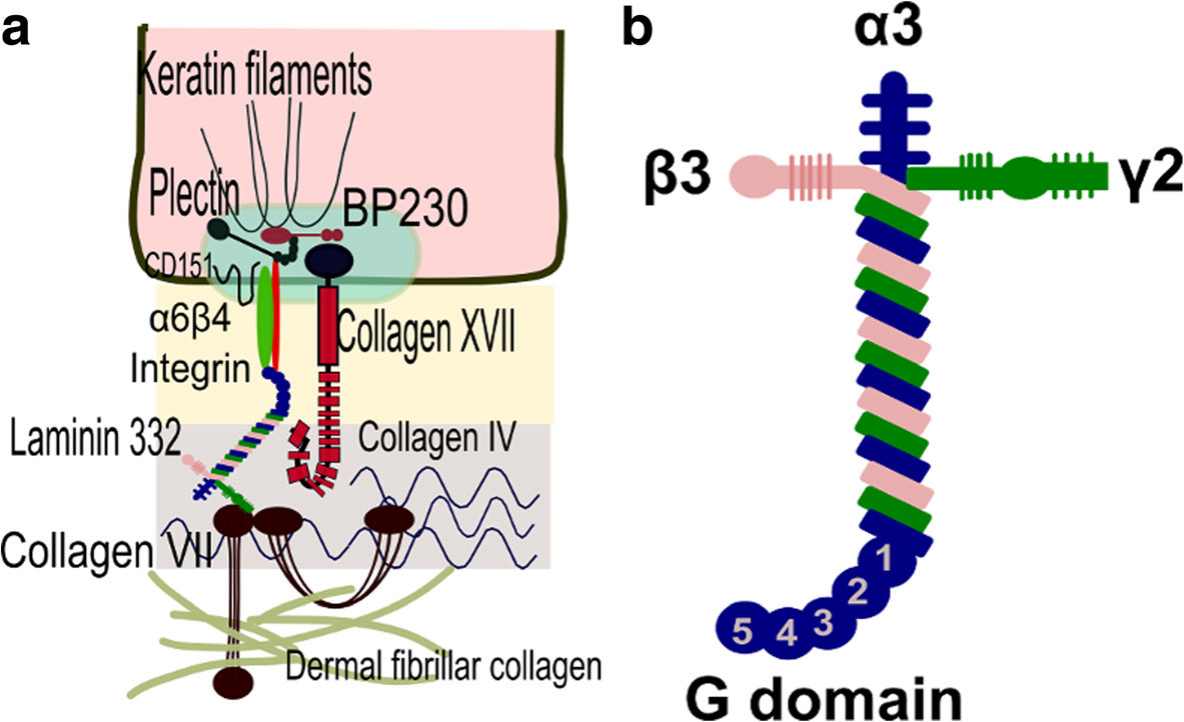
Localisation and structure of laminin 332 at the dermo-epidermal junction. **a** Laminin 332, a major component of the lamina densa, is an extracellular matrix protein binding to collagen XVII/BP180, α6β4 integrin, and collagen VII. **b** Laminin 332 is a heterotrimer composed of α3, β3, and γ2 chains showing a cross-like structure with a large globular domain at the base of the cross

Laminin-332 (also known as laminin-5, epiligrin, nicein, ladsin, or kalinin) is an essential component of the epithelial basement membranes and is synthesized by keratinocytes. It has an important role in mediating dermal-epidermal adhesion, regeneration and repair of wounded skin. At the ultrastructural level, it appears as a cross-like structure with a large globular domain at the base of the cross, composed of α3, β3 and γ2 chains (Fig. 2) [17, 19]. Patients with anti-laminin-332 mucous membrane pemphigoid show autoantibodies specific to α3, β3 or γ2 subunits of laminin 332 [20–23].

Despite its diagnostic and prognostic significance, immunoassays for the detection of autoantibodies against laminin 332 have not yet been established in the clinical laboratory routine on a broad basis [1, 17]. The immunoprecipitation of radiolabelled cultured keratinocytes has been the golden standard for the detection of laminin 332-specific autoantibodies in patients with mucous membrane pemphigoid [6, 24]. Alternatively, autoantibodies against laminin 332 may be detected by immunoblotting using extracellular matrix of cultured human keratinocytes or purified laminin 332 [23]. These qualitative immunoassays are time-consuming, laborious and thus restricted to a few specialized laboratories worldwide. In addition, the use of radioimmunoassays, although highly sensitive and specific, is laborious, expensive and tightly regulated [6, 20, 21, 25, 26]**.** In addition, ELISA systems for the detection of laminin 332-specific autoantibodies using purified native laminin 332 [27, 28] and keratinocyte extracellular matrix [18] have been described. However, these testing ELISA systems also show several limitations. In the two studies performed by Bekou et al. [27] and Bernard et al. [28] using affinity purified native laminin 332, patients sera were not tested against other established methods of detecting laminin 332-specific autoantibodies. A third study tested a number of only 32 MMP patients for laminin 332-specific autoantibodies by ELISA using as substrate extracellular matrix from normal human keratinocytes [18].

Therefore, the aim of this study was to investigate the diagnostic suitability of several molecular immunossays using native and recombinant laminin 332 as well as the autoantigen-rich HaCaT keratinocyte extracellular matrix. While all ELISA systems tested in our study performed robustly, we emphasize the potential significance of the quantitative immunoassay using the extracellular matrix of the immortalised keratinocyte as an adjuvant tool in the diagnostic algorithm of this rare and elusive pemphigoid variant.

## Methods

### Human sera

Serum samples were obtained from patients with mucous membrane pemphigoid (MMP; *n* = 200), bullous pemphigoid (BP; *n* = 89), pemphigus vulgaris (PV; *n* = 49), epidermolysis bullosa acquisita (EBA; *n* = 19), dermatitis herpetiformis (DH; *n* = 41) and healthy donors (*n* = 116). The inclusion criteria of the patients closely followed the currently accepted diagnostic criteria for bullous autoimmune diseases [1, 29]. The inclusion criteria for MMP patients (n = 200) were: 1) involvement of mucous membranes, including erosions, blisters or scarring of ocular, oral, laryngeal, esophageal, genital mucosa and 2) presence of linear IgG and/or IgA and C3 deposits, along the basement membrane of perilesional mucosa and/or skin. Within the MMP group, we selected a subgroup of “confirmed anti-epiligring MMP” patients (*n* = 36) which tested positive for laminin 332-specific autoantibodies by immunoblotting with purified native autoantigen or extracellular matrix of cultured keratinocytes. BP patients were characterized by: (a) subepidermal skin blisters, (b) linear IgG deposits along the dermoepidermal junction detected by direct IF microscopy, (c) circulating IgG autoantibodies binding to the epidermal side of the salt-split skin as revealed by indirect IF microscopy and (d) presence of autoantibodies against collagen XVII/BP180-NC16A or BP230. PV patients were characterized by: (a) intraepidermal skin blisters and mucosal or mucocutaneous involvement, (b) intercellular IgG deposits within the epidermis detected by direct immunofluorescence (DIF) microscopy, (c) serum IgG autoantibodies binding to the epithelium of monkey esophagus with an intercellular pattern by indirect IF microscopy, and (d) IgG autoantibodies against desmoglein 3 and desmoglein 1 by ELISA. EBA patients were characterized by: (a) subepidermal skin blisters, (b) linear IgG deposits along the dermoepidermal junction detected by direct IF microscopy, (c) circulating IgG autoantibodies binding to the dermal side of the salt-split skin as revealed by IF microscopy and (d) presence of collagen VII-specific autoantibodies as revealed by ELISA. DH patients were characterized by (a) chronic subepidermal blistering skin disease characterized by pruritic papulo-vesicular lesions, (b) granular IgA deposits at the epidermal basement membrane by direct IF microscopy, (c) anti-endomysial IgA antibodies by indirect IF microscopy on monkey esophagus and IgA specific for epidermal/tissue transglutaminase by ELISA. Serum samples were collected at diagnosis, before initiation of therapy and stored at − 80 °C until analysis. The study was approved by the local Ethic Committee and performed upon informed consent in accordance with the Declaration of Helsinki.

### Generation of laminin 332-rich extracellular matrix of immortalised HaCaT keratinocytes

HaCaT cells were cultured in DMEM medium (Life Technologies), supplemented with 10% FCS, L-glutamine and 1% penicillin-streptomycin. When overconfluent, cells were washed 3× with PBS and removed from the flask surface, by 10 min incubation at 37 °C, with 5 ml 1:10 diluted Trypsin (Biochrom, Catalog No. L2153), then 3 ml FCS (PAA Laboratories GmbH, Catalog No. A11–151), for neutralization. Subsequently, cells were centrifuged for 10 min, at room temperature, then resuspended in 20 ml DMEM medium, supplemented with 10% FCS, L-glutamine and 1% penicillin-streptomycin and transferred to a BioGreiner flat, high-binding ELISA plate (Bio Greiner, Catalog no: 655101), resuspended in 200 μl medium/well. When overconfluent, cells were washed 3× with 300 μl PBS/well, then removed after incubation under the microscope, using 20 mM NH4OH solution, 100 μl/well. The extracellular matrix was washed with 3× MiliQ water, 300 μl/well and 3× PBS, 300 μl/well. ELISA was then performed on the extracellular matrix still adhering on the well surface.

### Generation of recombinant and native forms of laminin 332

Differently glycosylated forms of laminin 332 were purified from the serum-free conditioned media of GnT-III- or GnT-V-overexpressing MKN45 transfectants as described previously [30]. Briefly, the collected media were precipitated by 80% saturated ammonium sulfate. The precipitate was dissolved in and dialysed against a buffer containing 20 mM Tris-HCl (pH 7.5), 0.1 M NaCl, 0.1% CHAPS, 0.005% Brij 35. The sample was precleared by a centrifugation at 19,000 rpm for 30 min at 4 °C and then passed through a gelatin column. The flow-through of a gelatin column was directly applied to a laminin α3-specific antibody column and then laminin 332 was eluted by 0.05% trifluoroacetate (*v*/v), which was neutrized with Tris-HCl (pH 8.0) containing 0.005% Brij 35 and 0.1% CHAPS.

### Sodium dodecylsulfate polyacrylamide gel electrophoresis (SDS-PAGE) and immunoblot analysis

**Fig. 3:**
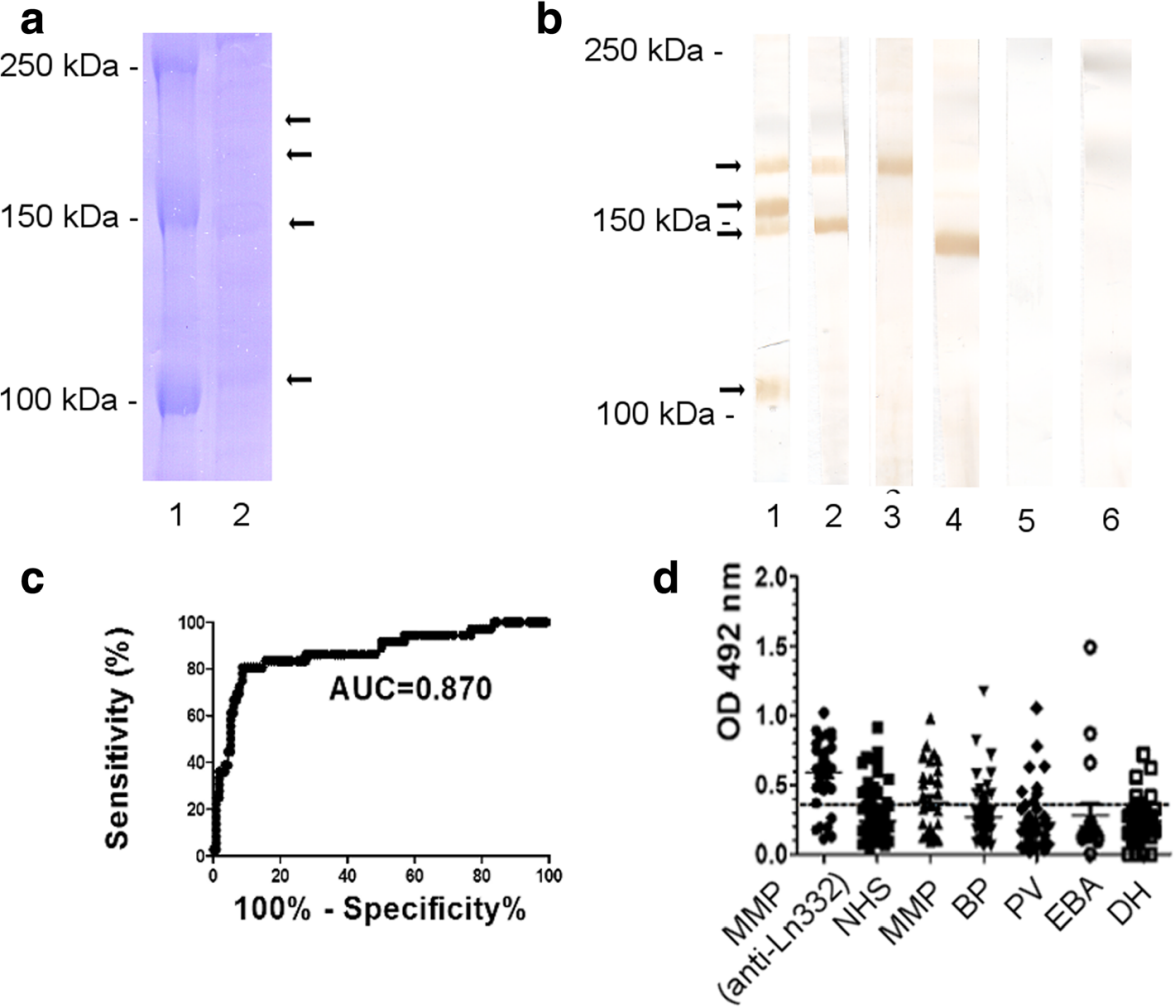
Detection of autoantibodies by ELISA with extracellular matrix of cultured keratinocytes. The extracellular matrix was obtained by treatment of cultured HaCaT keratinocytes with 20 mM NH4OH as detailed in Methods. **a** SDS-PAGE analysis of the extracellular matrix extract shows the migration of laminin 332 chains (arrows) at about 165 kDa (α3 chain), 140 kDa (β3 chain) as well as 155 kDa and 105 kDa (unprocessed and processed forms of the γ2 chain). **b** Immunoreactivity of mucous membrane pemphigoid autoantibodies with the laminin 332 from the extracellular matrix extract. Extracellular matrix extract was electrophoretically separated by 8% SDS-PAGE, transferred to nitrocellulose and immunoblotted with rabbit anti-human laminin 332 polyclonal antibody (lane 1), anti-laminin 332 mucous membrane pemphigoid patient’s sera (lanes 2–4), normal human sera (lane 5), and normal rabbit sera (lane 6). **c** Receiver-operating-characteristic (ROC) curve. AUC, area under the curve. Test performed with sera from patients with confirmed anti-laminin 332 mucous membrane pemphigoid (*n* = 36) and controls (*n* = 116). **d** ELISA reactivity of human sera with extracellular matrix. Scatter plots represent optical density measurements of serum reactivity from patients with mucous membrane pemphigoid (MMP; n = 36) with confirmed laminin 332-specific autoantibodies, MMP patients with possible laminin 332-specific autoantibodies (*n* = 30), from healthy donors (NHS; n = 116), as well as from patients with bullous pemphigoid (BP; *n* = 89), pemphigus vulgaris (PV; *n* = 49), epidermolysis bullosa acquisita (EBA; *n* = 19), and dermatitis herpetiformis (DH; *n* = 41). The cut-off of the assay is represented by a dotted line (cut-off = 0.367, sensitivity = 83.33%, specificity = 84.48%)

Immunoblotting with extracellular matrix extract from HaCaT cells, and GnT-III- and GnT-V-laminin 332 were performed as previously described with minor modifications [23, 30]. Briefly, proteins were separated by SDS-PAGE on 8% preparative gels, under reducing conditions, followed by transfer onto nitrocellulose (Whatman/Protran BA85). Membrane strips were blocked in 5% skimmed milk, for one hour, at room temperature. Then, strips were rinsed in 1× TBS-Tween and incubated for 2 h with 200-fold diluted MMP sera as well as normal human and rabbit sera and human laminin 332-specific rabbit polyclonal antibody (5LN9) as controls. Strips were washed 3×, 10 min in 1× TBS-Tween. Then, strips were incubated for 1.5 h with a secondary 2000-fold diluted, HRP-conjugated goat anti-human IgG antibodies (Abcam, Catalog no: ab6858) and HRP-conjugated chicken anti-rabbit IgG antibodies (NOVUS biologicals, Catalog no: NB120–6829). After washing 3× with TBS-Tween, reactivity was visualized with diaminobenzidine (Merck, Catalog no:1029240001). When separated by SDS polyacrylamyde gel, the extracellular matrix extract showed the migration of laminin 332 chains at about 165 kDa (α3 chain), 140 kDa (β3 chain) and 155 kDa and 105 kDa (γ2 chain, unprocessed and processed forms) (Fig. 3a). The immunoreactivity of the laminin 332 from the extracellular matrix from cultured HaCaT cells was analyzed by immunoblotting with specific polyclonal rabbit antibody, sera from reference MMP patients and healthy donors. IgG autoantibodies from anti-laminin 332 MMP patients, but not from healthy donors recognized the chains of the laminin 332 protein (Fig. 3b).

### Indirect IF microscopy

Presence of circulating IgG autoantibodies was detected by indirect IF microscopy, following published protocols [31]. Briefly, frozen sections of salt-split skin were incubated in a first step with 10- or 20-fold diluted sera. IgG antibodies bound at the epidermal basement membrane were visualized using 100-fold diluted, Alexa Fluor488-labelled polyclonal goat anti-human IgG antibody (Life Technologies). Bound IgG4 autoantibodies were visualised by incubating the section with a 100-fold diluted biotin-labelled human IgG4-specific monoclonal antibody (Invitrogen) and subsequently with AlexaFluor488-conjugated streptavidine (Life Technologies).

### Enzyme-linked immunosorbent assay (ELISA) for detection of anti-BP180-NC16A autoantibodies

In order to determine levels of collagen XVII/BP180-specific autoantibodies at diagnosis, we have used commercially available ELISA kits (MBL laboratories, Nagoya, Japan) [32]. The cut-off value for positive levels of anti-BP180-NC16A antibodies was considered as ≥9 U/mL.

### Development of ELISA for detection of laminin 332-specific autoantibodies using HaCaT extracellular matrix

The plates were washed 5× with 0.05% Tween20-PBS (*w*/*v*) and blocked 1.5 h with 5% BSA-PBS (w/v) followed by incubation with 1:100 diluted sera in 1% BSA-0.05% Tween20-PBS (w/v) for 1.5 h. Plates were washed 5× with 0.05% Tween20-PBS (w/v). Bound antibodies were detected by 1.5 h incubation with 2000-fold diluted horseradish-peroxidase goat antihuman IgG antibodies (Abcam, Catalog no: ab6858). After washing 5× with 0.05% Tween20-PBS (w/v), color reaction was developed by addition of orthophenylene diamine substrate (Dako, Catalog No: S2045). Reaction was stopped after 10 min with 0.5 M sulfuric acid solution. All steps were carried out at room temperature. The optical density (OD) was read at 492 nm using an automated spectrophotometer (BioTek,MWGt, Sirius HT-TRF,Gen5 programme, Version 2.01). Each serum was tested in duplicate. The cut-off for positivity was validated and optimized by receiver-operating characteristics (ROC) analysis as described below. The accuracy of the assay was expressed as sensitivity = true positive/ (true positive + false negative) and specificity = true negative/(true negative + false positive).

### ELISA using native and recombinant commercially available laminin 332

Native commercially available laminin 332 purified by affinity chromatography from cell culture supernatant of human foreskin keratinocytes has been purchased from Immundiagnostik. Human recombinant commercially available laminin 332, produced by human HEK293 cells transfected with human laminin-332 genes and purified by a series of fast protein liquid chromatography procedures, according to the manufacturer’s protocols has been obtained from Biolamina (Product Number: LN332–02, Batch Number: 80091). ELISA was developed and performed using previously established protocols with modification [33, 34]. Briefly, 96-well microtiter plates with flat bottom (Greiner Bio-One, Germany) were coated with 400 ng/well of native or recombinant laminin 332 in 0.05 M bicarbonate buffer (pH 9.6), overnight at 4 °C. Next day the plates were washed 3× with 0.05% Tween20-PBS (*w*/*v*) and blocked 1 h with 2% BSA-PBS (w/v) followed by incubation with 1:100 diluted sera in 1% BSA-0.05% Tween20-PBS (w/v) for 1 h. Plates were washed again with 3× with 0.05% Tween20-PBS (w/v). Then, bound antibodies were detected by 1 h incubation with 2000-fold diluted horseradish-peroxidase goat antihuman IgG antibodies (Abcam, Catalog no: ab6858). After washing, color reaction was developed by addition of orthophenylene diamine substrate (Dako, Catalog No S2045). Reaction was stopped after 10 min with 0.5 M sulphuric acid solution. All steps were carried out at room temperature. The optical density (OD) was read at 492 nm using an automated spectrophotometer (BioTek,MWGt, Sirius HT-TRF,Gen5 programme, Version 2.01). Each serum was tested in duplicate. The cut-off for positivity was validated and optimized by receiver-operating characteristics (ROC) analysis. The accuracy of the assay was calculated as sensitivity = true positive/(true positive + false negative) and specificity = true negative/(true negative + false positive).

### Statistical analysis

Statistical analysis has been performed using GraphPad Prism 5.03 (https://www.graphpad.com/scientific-software/prism/), QtiPlot (http://www.qtiplot.com/) and the R statistical package (https://www.r-project.org/).

## Results

### Characterization of laminin 332-specific autoantibodies in MMP patients

In our present study, we have stratified the MMP patients based on their serum reactivity and the respective molecular specificity of the autoantibodies. A first subgroup of MMP (*n* = 36), included patients with confirmed laminin 332-specific antibody reactivity as detected by immunoblotting using extracellular matrix of cultured keratinocytes (Fig. 3a, b). A second subgroup included MMP patients (*n* = 26) with possible laminin 332-reactivity defined based on the following criteria: (1) presence of anti-laminin 332 autoantibodies by immunoblotting using commercially available laminin 332; (2) lack of binding of IgG autoantibodies to the epidermal side by indirect IF microscopy on 1 M NaCl salt split skin; (3) lack of IgG anti-BP180-NC16A autoantibodies by ELISA; (4) lack of IgG anti-collagen VII autoantibodies by immunoblotting using NC1-hCVII. A further subgroup included MMP patients (*n* = 128) with other disease variants. In addition, we have used sera from healthy donors (*n* = 116).

**Fig. 4:**
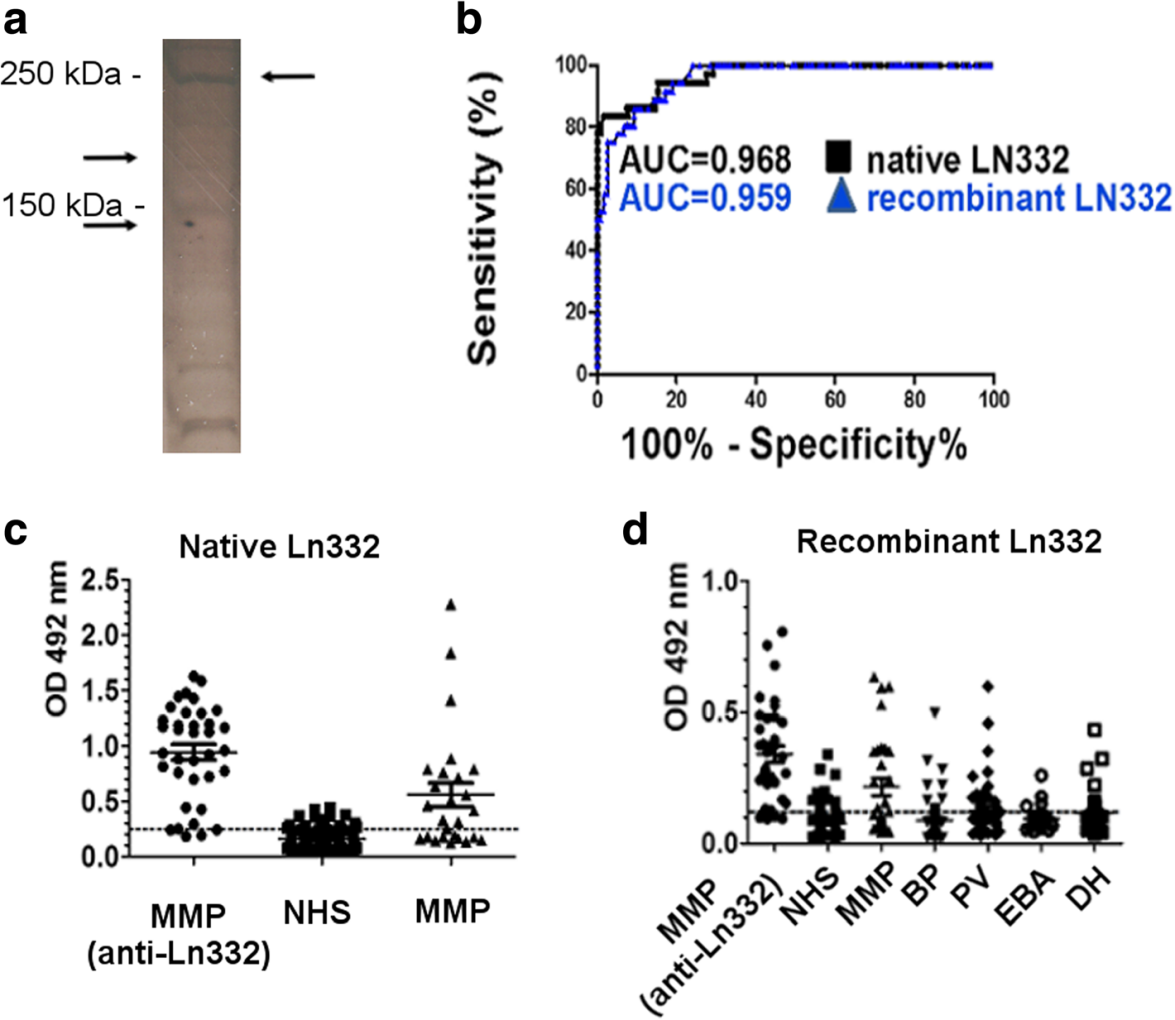
Comparative reactivity of patients’ sera with native and recombinant laminin 332 by ELISA. **a** Silver staining of the polyacrylamide gel of the reduced recombinant laminin 332 shows the migration of recombinant laminin 332 at about 260 kDa (β3 chain, 129KDa and γ2 chain, 130KDa). **b** Receiver-operating-characteristic (ROC) curve. AUC, area under the curve. Test performed with sera from patients with confirmed anti-laminin 332 mucous membrane pemphigoid (n = 36) and controls (n = 116). AUC for native laminin 332 = 0,968 (shown in black). AUC for recombinant laminin 332 = 0,959 (shown in blue). **c** ELISA reactivity of human sera with the native laminin 332. Scatter plots represent optical density measurements of serum reactivity of confirmed anti-laminin 332 mucous membrane pemphigoid (MMP) (n = 36), normal human sera (n = 116), and possible anti-laminin 332 (*n* = 26). The cut-off of the assay is represented by a dotted line (cut-off = 0.242, sensitivity = 94.44%, specificity = 84.48%). **d** ELISA reactivity of human sera with recombinant laminin 332. Scatter plots represent optical density measurements of serum reactivity of confirmed anti-laminin 332 mucous membrane pemphigoid (MMP) (n = 36), normal human sera (n = 116), possible anti-laminin 332 (n = 26), bullous pemphigoid (BP, n = 116), pemphigus vulgaris (PV, n = 116), epidermolysis bullosa acquisita (EBA, n = 116) and dermatits herpetiformis (DH, n = 116). The cut-off of the assay is represented by a dotted line (cut-off = 0.103, sensitivity = 91.67%, specificity = 82.76%)

Sera with laminin 332-specific reactivity confirmed by immunoblotting were further tested by ELISA using purified, native autoantigen. Under these conditions, the subsequent ROC analysis showed an area under the curve of 0.968 (95% CI: 0.9418 to 0.995; *p* < 0.0001) (Fig. 4b, AUC shown in black). Based on these results, we have chosen a cut-off OD value of 0.242 OD reading units, with a calculated sensitivity of 94.44% (95% CI: 81.34–99.32%) and specificity of 84.48% (95% CI: 76.59–90.54%). In this MMP subgroup, 34 of 36 (94.44%) patients with confirmed laminin 332-specific autoantibodies showed positive reactivity by ELISA. In addition, 16 of 26 (61.53%) MMP patients with possible anti-laminin 332-specific autoantibodies demonstrated positive reactivity with the substrate at the chosen cut-off. A total of 74 of 128 (57.81%) of MMP patients with other disease variants showed positive reactivity in the immunoassay. Also 18 of 116 (15.52%) sera from healthy donors showed positive results in this ELISA (Fig. 4c).

### Detection of IgG autoantibodies by ELISA with native and recombinant laminin 332

In a first set of experiments, we investigate the suitability of native and recombinant forms of laminin 332 for the detection of specific autoantibodies. For this purpose, we have performed the immunoassay using the same coating antigen amount for both forms of laminin 332. As a reference positive group, we have used the MMP sera (*n* = 36) with confirmed laminin 332-specific reactivity. In addition, we tested MMP patients with possible laminin 332-specific reactivity (*n* = 30) and with other MMP variants (*n* = 134) as well as patients with bullous pemphigoid (BP, *n* = 89), pemphigus vulgaris (PV, *n* = 49), epidermolysis bullosa acquisita (EBA, *n* = 19), and dermatitis herpetiformis (DH, *n* = 41). As negative reference group, we have used sera from healthy donors (*n* = 116). The area under the ROC curve for recombinant laminin 332 was 0.959 (95% CI:0.931 to 0.987; *p* < 0.0001) (Fig. 4b, AUC shown in blue). Based on these findings, we have chosen a cut-off OD value of 0.103 OD reading units, with a calculated sensitivity of 91.67% (95% CI: 77.53 to 98.25%) and specificity of 82.76% (95% CI: 74.64 to 89.14%). Under these conditions, 33 of 36 (91.67%) MMP patients with confirmed laminin 332-specific autoantibodies and 17 of 30 (56.66%) MMP patients with possible laminin 332-specific autoantibodies showed positive reactivity in ELISA. In addition, 68 of 134 (50.74%) patients with other variants of MMP showed positive reactivity. Also, 20 of 116 (17.24%) healthy donors showed positive results in ELISA. These results yielded an AUC of 0.968 for native laminin 332 (Fig. 4b, in black) and of 0.959 for recombinant laminin 332 (Fig. 4b, in blue). In the additional control groups, 23 of 89 (25.84%) BP patients, 23 of 49 (46.93%) PV patients, 7 of 19 (36.84%) EBA patients and 16 of 41 (39.02%) DH patients tested positive in the immunoassay (Fig. 4d).

### Detection of autoantibodies by ELISA using laminin 332-rich keratinocyte extracellular matrix

To develop an immunoassay for the detection of autoantibodies using the extracellular matrix of keratinocytes we have used sera from MMP patients with confirmed laminin 332 autoreactivity (*n* = 36) and from healthy controls (*n* = 116). The ROC analysis showed an area under the curve of 0.87 (95%CI: 0.794 to 0.945; *p* < 0,0001) (Fig. 3c). Based on this, we established a cut-off value of 0.367 yielding a sensitivity of 83.33% (95%CI: 67.19 to 93.63%) and a specificity of 84.48% (95%CI: 76.59 to 90.54%). Using the optimised conditions, we have subsequently tested sera from further patients’ groups, including MMP patients with possible laminin 332-specific autoantibodies (*n* = 30), other MMP variants (*n* = 134), patients with BP (*n* = 89), PV (*n* = 49), EBA (*n* = 19), and DH (*n* = 41). Thirty of 36 (83.33%) MMP patients with confirmed laminin 332-specific autoantibodies showed positive reactivity in ELISA. Results for the other groups showed that 13 of 30 (43.33%) and 37of 134 (27.16%) patients with possible laminin 332-specific autoantibodies and other MMP variants, respectively, reacted positive in this immunoassay. Also 18 of 116 (15.51%) healthy donors showed positive results in ELISA. In addition, 15 of 89 (16.85%) BP patients, 8 of 49 (16.32%) PV patients, 3 of 19 (15.78%) EBA patients, and 5 of 41 (12.19%) DH patients showed positive results (Fig. 3d).

ELISA using extracellular matrix from HaCaT cells showed a lower AUC, compared with ELISA using native and recombinant laminin 332 (AUC = 0.870 for ELISA using extracellular matrix from HaCaT cells, compared to AUC = 0.968 for native laminin 332 vs. AUC = 0.959 for recombinant laminin 332).

### Autoantibodies from BP patients do not bind to the lamina densa of the epidermal basement membrane

**Fig. 5:**
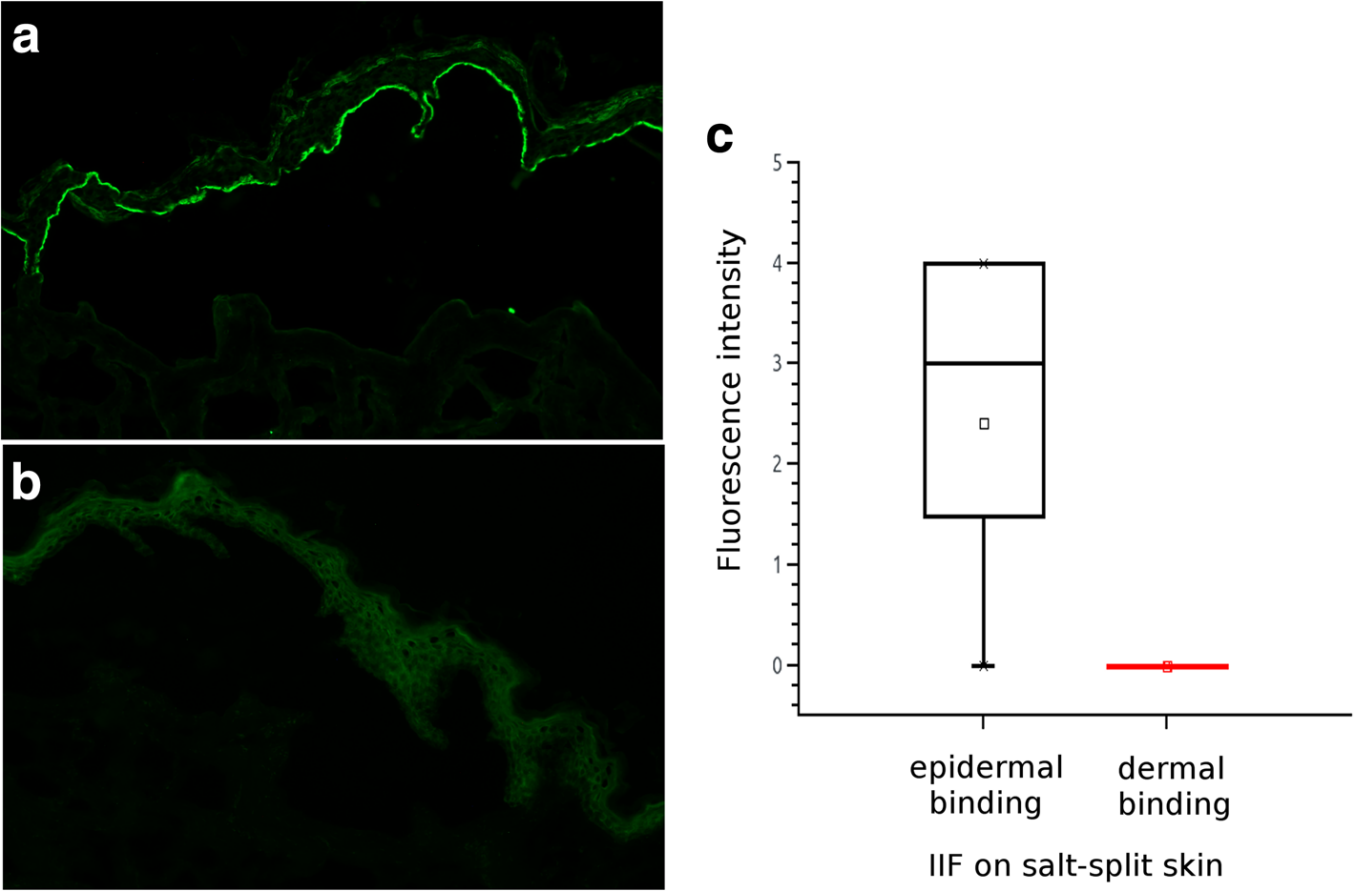
Reactivity of sera from patients with bullous pemphigoid (BP) by indirect immunofluorescence microscopy. Sera of patients with BP (*n* = 88) were incubated with sections of human split-skin. Binding of autoantibodies was visualised by using a human IgG4-specific biotinylated secondary monoclonal antibody and AlexaFluo448-labelled streptavidin. The fluorescence intensity was evaluated on a scale from 0 (no binding) to 4 (maximum). Binding of IgG4 autoantibodies from a (**a**) patient with BP and (**b**) a healthy donor by indirect immunofluorescence. **c** Box-plot of epidermal vs. dermal binding of serum autoantibodies in patients with BP

Our own present data and results of previous studies showed that sera from patients with control groups, including BP patients, showed positive reaction with purified native and recombinant laminin 332 preparations as well as with the laminin 332-rich extracellular matrix. To investigate a possible reactivity of BP sera (*n* = 88) with laminin 332, we have tested their IgG4 reactivity on sections of human split-skin by indirect IF microscopy. To avoid the background staining on organ section seen with a polyclonal secondary antibody, we have used a monoclonal antibody specific to human IgG4. Similar to other autoimmune bullous diseases, IgG4 autoantibodies are the predominant IgG isotype also in patients with BP and MMP. Human salt-split skin is obtained by incubation of normal human skin in a 1 M NaCl solution, which consistently generates a cleavage within the lamina lucida of the epidermal basement membrane. Extensive antigen mapping studies have clearly shown that laminin 332 always localises to the dermal side of the artificial cleavage. Our analysis using this robust assay showed that IgG4 autoantibodies from BP patients do not bind to the dermal-side of the split, while most of them show a strong binding to the epidermal side of the cleavage (Fig. 5). We extended our analysis using sera from patients with dermatitis herpetiformis and pemphigus vulgaris, which as expected also did not react with the dermal side on salt-split skin by indirect IF microscopy (data not shown). To further investigate the reactivity of human sera with laminin 332 preparations, we tested serum samples from patients with pemphigus vulgaris (*n* = 20), dermatitis herpetiformis (n = 20) and epidermolysis bullosa acquisita (n = 20) comparatively by ELISA using native purified laminin 332 and the extracellular matrix of keratinocytes as substrates (Additional file 1: Figure S1).

### Bisecting GlcNAc and β1,6GlcNAc residues of laminin-332 do not affect autoantigen recognition by MMP autoantibodies

**Fig. 6:**
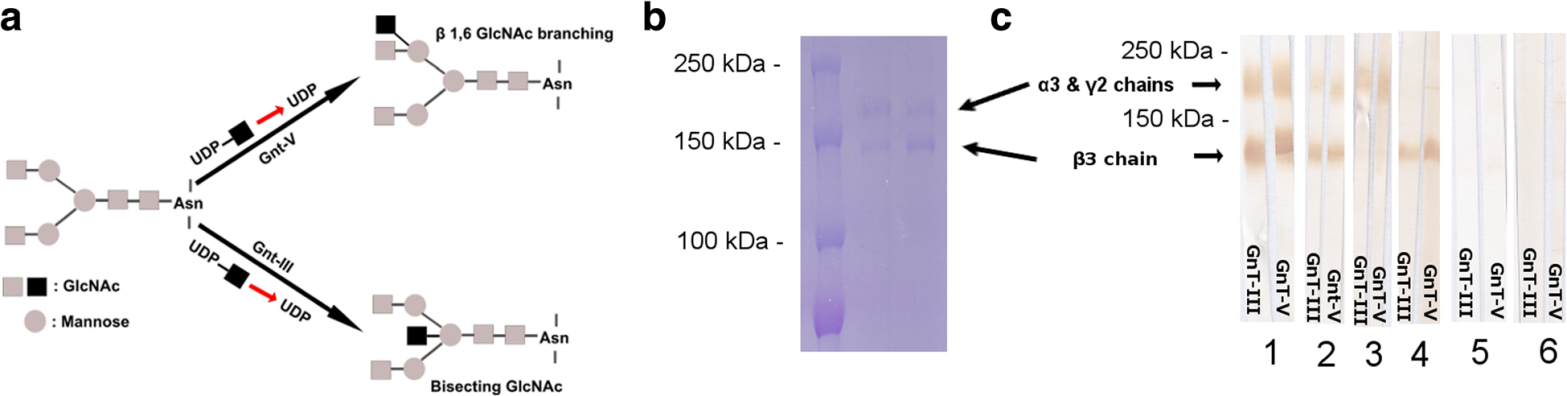
Immunoreactivity of IgG anti-laminin 332 autoantibodies with the GnT-III and GnT-V forms of laminin 332. **a** Schematic representation of GnT-III and GnT-V laminin 332. **b** Sodium dodecylsulfate-polyacrylamide gel electrophoresis (SDS-PAGE) of the GnT-III (lane 2) and GnT-V (lane 3) proteins shows migration of α3 and γ2 chains at around 160 kDa and β3 chain at around 135 kDa. Weight markers are shown in lane 1. **c** Immunoreactivity of mucous membrane pemphigoid autoantibodies with the GnT-III and GnT-V isoforms of laminin 332. The two isoforms of laminin 332 were electrophoretically separated by 8% SDS-PAGE, transferred to nitrocellulose and immunoblotted with rabbit anti-human laminin 332 polyclonal antibody (lane 1), anti-laminin 332 mucous membrane pemphigoid patients‘sera (lanes 2–4), normal human sera (lane 5) and normal rabbit sera (lane 6). Anti-laminin 332 autoantibodies reacted in a similar manner with the two isoforms of laminin 332 (GnT-III and GnT-V)

Previous studies showed that posttranslational modifications of autoantigens such as phosphorylation and deglycosylation may alter the binding of autoantibodies in pemphigoid diseases [35, 36]. It has also been shown that glycosylation might influence the laminin 332 functions in tumorigenesis such as tumour invasion or tumour suppression [37]. We cannot exclude the fact that these glycosylations may influence the binding of specific IgG autoantibodies from anti-laminin-332 MMP patient sera to the protein. In order to investigate this issue, we have performed a comparative immunoblot, using the two differently glycosylated forms of laminin 332, GnT-III- and GnT-V-laminin-332, which are highly modified with bisecting GlcNAc and β1,6GlcNAc, respectively (Fig. 6a) [37]. The migration of the two proteins in SDS-PAGE is shown in Fig. 6b. We have tested a number of 23 MMP sera. We did not observe a different binding of IgG autoantibodies to laminin-332 by immunoblotting, in the two proteins, as the MMP sera showed the same reactivity to both GnT-III- and GnT-V-laminin-332 (Fig. 6). From the tested sera, 14 were positive for both α3 and β3 chains of both GnT-III- and GnT-V-laminin-332, 4 were positive only for the α3 chain, 3 were positive only for the β3 chain, while 2 were negative for both chains (Fig. 6c).

## Discussion

The diagnosis of the rare and elusive anti-laminin 332 MMP poses serious difficulties in the clinical routine. Diagnostic criteria include the detection of tissue-bound autoantibodies by direct IF microscopy as well as the detection of autoantibodies and characterization of their molecular specificity by serological assays, including indirect IF microscopy, immunoprecipitation, immunoblot and ELISA. However, due to a low reactivity up to 50% of the MMP patients do not show autoantibody binding by indirect IF microscopy. In addition, while immunoprecipitation and immunoblotting are sensitive and specific, they are are non-quantitative, laborious and restricted to a few specialized laboratories worldwide. In addition, the use of radioimmunoassays, although highly sensitive and specific, is laborious, expensive and tightly regulated. The use of quantitative immunoassays for the detection of autoantibodies against laminin 332 has been attempted in a few previous studies, but due to several limitations these ELISA systems did not reach the stage of broad clinical diagnostic use so far. Therefore, our present study was mainly aimed at facilitating establishing quantitative immunoassays for the detection of laminin 332-specific antibodies as routine clinical diagnostics. Our comparative analysis of several immunoassays using different preparations of the autoantigen show their suitability as ancillary tools in the diagnostic algorithm for MMP and emphasize the use of the laminin 332-rich extracellular matrix of keratinocytes as an alternative, inexpensive substrate for these immunoassays.

In a first series of experiments, we have thoroughly characterized the autoantibody specificity in a group of MMP patients by immunoblotting with purified, native laminin 332. Since 1992, when the anti-epiligrin cicatricial pemphigoid has been described for the first time [6], immunoprecipitation using radioactive-labelled keratinocytes represented the gold standard for the detection of laminin 332-specific autoantibodies. Subsequent studies showed that immunoblotting using as substrate purified native laminin 332 [22] or keratinocyte extracellular matrix extract [23] is an equivalent to immunoprecipitation. Therefore, we have used the immunoblotting as reference method to define a set of sera with confirmed autoantibody reactivity to laminin 332.

Enzyme immunoassays such as ELISA have several advantages over other immunoassays, including indirect IF microscopy on organ sections, immunoprecipitation and immunoblotting. Thus, ELISA systems allow for obtaining numeric results, are less laborious and amenable to automation also in random-access analyzers. Therefore, in a next set of experiments, we have addressed the suitability of ELISA using purified native and recombinant laminin 332 forms to detect specific autoantibodies in the defined patients’ groups. Initial testing of MMP sera by ELISA with the purified, native laminin 332 yielded a high correlation with the results by immunoblotting. In further experiments, the reactivity of MMP sera and several other control groups were tested comparatively by ELISA using the purified, native and the recombinant form of the autoantigen. The results showed that both ELISA systems are robust assay highly sensitive and relatively specific. Our data are in line with the results of the two previous studies using purified native laminin 332 as antigenic substrate [27, 28] and show that recombinant laminin 332 is an equivalent substrate for the immunoassay. Extending and optimizing previous studies, we have strived to define a relatively large group of MMP patients by their positivity for laminin 332-specific autoantibodies by immunblotting with the purified, native autoantigen.

One previous study described the use of extracellular matrix of normal human keratinocytes as the antigenic substrate in ELISA for the detection of autoantibodies in MMP patients [18]. Although obtaining the extracellular matrix may first appear laborious, it is in fact a straightforward procedure, unexpensive and easy to perform for every laboratory where mammalian cell cultures belong to daily routine. To further optimize this system, we have now used immortalised keratinocytes, which are easy to obtain and maintain for undefinite time in culture compared with the generation and short-term passaging of primary human keratinocytes. In addition, the availability of the donor skin (e.g., neonatal foreskin) may be restricted by shortage and/or regulatory issues. Moreover, culturing primary keratinocytes is possible for only a few passages and the outcome depends more heavily on external factors and culture conditions. Our results indicate that ELISA with HaCaT extracellular matrix correlates well with the other quantitative immunoassays using native and recombinant autoantigen forms and shows a relatively high sensitivity. Although not specifically tested in the present study, preservation of the ELISA plates coated with extracellular matrix frozen or in the refrigerator appears feasible and should be addressed in future studies.

A common perturbing feature of the ELISA systems for detection of autoantibodies against laminin 332 is their relatively high percentage of positive sera from the control groups. This effect, which has been already documented by previous investigations [18, 27], does not appear to significantly depend on the used substrate as shown in the present study. One may only speculate that purified laminin 332 preparations could still contain laminin ligands, including the hemidesmosomal autoantigen BP180, or even intracellular proteins such as BP230. To further pursue this intriguing finding and answer the question whether BP sera show a “real” reactivity to laminin 332, we have tested the BP sera by indirect IF microscopy on split-skin. To increase sensitivity and specifity of this robust assay, he have used a fluorochrome-labelled human IgG4-specific monoclonal antibody to detect bound BP autoantibodies. In agreement with a previous study [18], our present results show that BP sera do not contain autoantibodies binding to the dermal side of the salt-split. The analysis of sera from patients with pemphigus, dermatitis herpetiformis and epidermolysis bullosa acquisita by ELISA with native laminin 332 and extracellular matrix confirmed previous findings that a significant proportion of these patients react with these substrates. The reactivity of sera from other disease groups and healthy donors may be labelled as “unspecific”, but is void of significance for the differential diagnosis of MMP.

Binding of specific autoantibodies to their target may be influenced by their posttranslational modifications of the antigen, as shown in previous studies also for collagen XVII/BP180 and pemphigoid autoantibodies [36]. Posttranslational modifications of laminin 332 could therefore be relevant for the recognition by MMP autoantibodies and thus for the development of an immunoassay. Therefore, in an attempt to address this question we have used two biologically relevant, differently glycosylated forms of laminin-332 [31]. Our immunoblot analysis using well-characterized MMP sera did not show any significant difference in their reactivity with both differently N-glycosylated forms of laminin 332 suggesting that glycosylation is not modulating the recognition of this antigen by autoantibodies.

In line with previous studies, our results show a good overall correlation between immunoblotting and ELISA systems using different laminin 332, with some deviations readily explainable by the methodological differences of the two immunoassays. Importantly, our study shows that not only native laminin 332, but also its recombinant form, both of which are commercially available, can be used for the detection of MMP autoantibodies. A third substrate explored in our study, the HaCaT extracellular matrix showed a somewhat slightly lower sensitivity and specificity compared to the ELISA using native and recombinant laminin 332. While this antigenic substrate is not yet commercially available, it may be easily and cheaply generated along the way in some university laboratories.

**Fig. 7:**
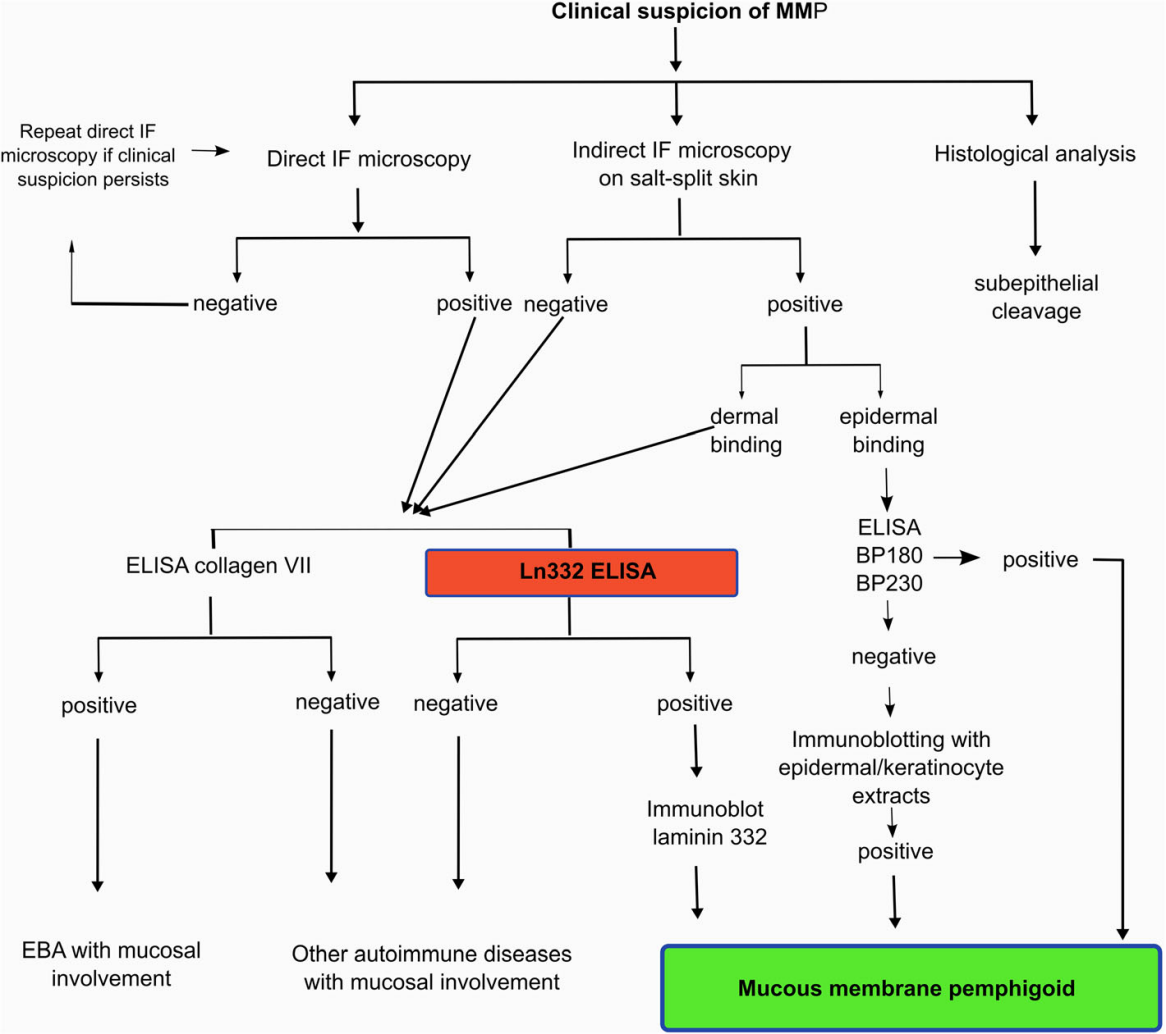
Use of laminin 332-specific immunoassays in the diagnostic algorithm in mucous membrane pemphigoid. The ELISA with extracellular matrix could represent an addition in patients with a high index of clinical suspicion of mucous membrane pemphigoid showing compatible results by direct immunofluorescence microscopy and either negative or positive findings with dermal binding by indirect immunofluorescence microscopy. A positive result by ELISA with keratinocyte extracellular matrix should be subsequently confirmed by immunoblotting

Our study using a high number of MMP sera with well-defined reactivity strongly suggests that immunoassays using laminin 332 may be a helpful addition to the diagnostic armamentarium in MMP. As detailed in Fig. 7, a possible diagnostic algorithm in MMP could include the ELISA with laminin 332 as the next step, especially in patients with dermal autoantibody binding or negative results by indirect IF microscopy. Positive findings by the ELISA should be confirmed by immunoblotting using HaCaT extracellular matrix or purified laminin 332.

## Conclusions

The salient feature of this study represents its more systematic approach to optimize the molecular diagnosis in MMP. Using a relatively large number of sera from MMP patients with well-characterized autoantibody reactivity we show the suitability of ELISA systems using laminin 332 preparations as adjunct diagnostic tools in MMP. While glycosylation of laminin 332 does not appear to influence its recognition by MMP autoantibodies, ELISA systems using both purified, native and recombinant laminin 332 demonstrated a high sensitivity and good correlation with the detection of autoantibodies by immunoblotting. These two commercially available laminin 332 preparations are valuable substrates for the quantitative detection of MMP autoantibodies. Despite a marginally lower diagnostic performance, the ELISA using the keratinocyte extracellular matrix represents a cheap, feasible alternative for university laboratories. The present data qualify the use of immunoassays using laminin 332 preparations as an additional diagnostic tool and facilitate further optimization of the diagnostic work-up in MMP.

## Additional file

Additional file 1: **Figure S1.** Comparative analysis of serum reactivity with the extracellular matrix and native laminin 332 by ELISA in control patients. Box plots represent optical density measurements of serum reactivity from patients with pemphigus vulgaris (PV, *n* = 20), epidermolysis bullosa acquisita (EBA, n = 20) and dermatits herpetiformis (DH, n = 20) as well as from healthy donors (*n* = 4) measured in parallel on native laminin 332 and laminin-rich extracellular matrix of keratinocytes. (PNG 20 kb)

## Abbreviations

AUC: Area under the curve;
BP: Bullous pemphigoid
BSA: Bovine serum albumine
DMEM: Dulbecco'’s Modified Eagle'’s medium
EBA: Epidermolysis bullosa acquisita
ELISA: Enzyme linked immunosorbent assay
FCS: Fetal calf serum
HaCaT: Calcium-induced Human Keratinocytes
HRP: Horseradish peroxidase
IB: Immunoblot
IF: Immunfluorescence
IgG: Immunoglobulin G
MMP: Mucous membrane pemphigoid
OD: Optical density
PBS: Phosphate-buffered saline
PV: Pemphigus vulgaris
ROC: Receiver-operating characteristics
TBS: Tris buffered saline.
