# Supplementary figures and images for: Molecular diagnosis of anti-laminin 332 (epiligrin) mucous membrane pemphigoid
Source: Orphanet J Rare Dis. 2018 Jul 6;13:111. doi: 10.1186/s13023-018-0855-x (PMC6035451; doi:10.1186/s13023-018-0855-x)

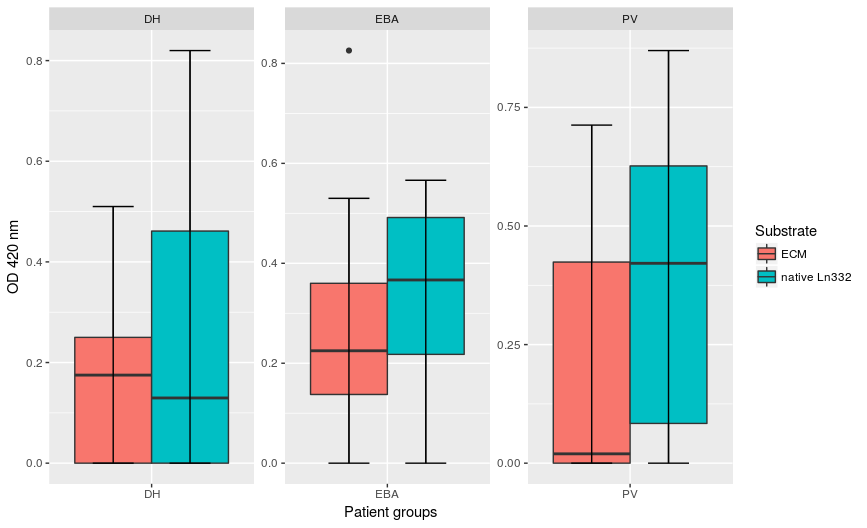

Supplement: Supplementary file 1 — Figure S1. Comparative analysis of serum reactivity with the extracellular matrix and native laminin 332 by ELISA in control patients. Box plots represent optical density measurements of serum reactivity from patients with pemphigus vulgaris (PV, n = 20), epidermolysis bullosa acquisita (EBA, n = 20) and dermatits herpetiformis (DH, n = 20) as well as from healthy donors (n = 4) measured in parallel on native laminin 332 and laminin-rich extracellular matrix of keratinocytes. (PNG 20 kb) [file 13023_2018_855_MOESM1_ESM.png]
